# No strain, no gain: within-host bacterial microdiversity and human health

**DOI:** 10.1128/iai.00062-26

**Published:** 2026-10-12

**Authors:** Anya Wojtkowiak, Melissa S. Shinfuku, Gina R. Lewin

**Affiliations:** 1 Center for Global Health and Diseases, Department of Pathology, Case Western Reserve University School of Medicine 12304, Cleveland, Ohio, USA; 2 Department of Molecular Biology and Microbiology, Case Western Reserve University School of Medicine 196211, Cleveland, Ohio, USA; 3 Case Western Reserve University-Cleveland VA Medical Center for Antimicrobial Resistance and Epidemiology 2546, Cleveland, Ohio, USA; University of California at Santa Cruz, Santa Cruz, California, USA

**Keywords:** population diversity, microdivesity, intra-species diversity, human microbiome, chronic infection, strain diversity

## Abstract

It is well established that within a bacterial species, genotypic and phenotypic variation leads to differences in virulence, antibiotic susceptibility, and metabolism. However, there is also increasing evidence that often multiple variants of the same species co-inhabit the same site in the human body, both during health and disease. This within-host microdiversity can develop over time for pathogens in chronic infections and has been observed for many members of the microbiota, for instance in the gut and on the skin. Research is just beginning to uncover how this within-host intra-species diversity develops and why it matters. Intra-species diversity is largely stable over time, with occasional selective sweeps of new genotypes, and the overall level of diversity is modulated by both microbe-microbe and microbe-host interactions. Furthermore, data indicate that within-host microdiversity can impact the stability of bacterial communities, their antimicrobial resistance, and the efficacy of probiotics and microbiota transplants. Here, we describe the evolutionary and ecological processes that promote high or low within-host microdiversity and the extent of this diversity in the human microbiota and in infections. In addition, we consider how microdiversity within individuals shapes the functional role of the human microbiota and the progression of disease. Together, within-host microdiversity is a newly developing way to think about the diversity of human-associated microbes, with implications in how we treat chronic infections and manipulate our microbiota to promote health.

## INTRODUCTION

The bacterial world is teeming with diversity, from cyanobacteria to episymbiotic Saccharibacteria to the well-studied *Escherichia coli*. However, even within a single species, organisms vary in their gene content and gene sequences, leading to significant functional differences across very closely related bacteria. For instance, two isolates of *E. coli* may share only 40% of the same gene content, allowing different strains to function as human pathogens, gut mutualists, or environmental specialists (1–4). Although this population-level diversity in bacteria has been long-recognized, recent studies have increasingly emphasized that a single person can harbor multiple genotypes of a species at the same time within a single body site. High within-host intra-species diversity can occur in the human microbiota, including in the gut, skin, oral cavity, and vagina. Furthermore, although acute infections are primarily monoclonal, chronic infections can often be polyclonal. Both in the microbiota and in chronic infections, this high intra-species diversity can occur when multiple strains colonize a site over time and when bacteria diversify through evolutionary processes.

Here, we review what is known about within-host bacterial intra-species diversity both in the healthy human microbiome and during human infections. To this end, we first define intra-species diversity and explore methods used to study intra-species diversity. We then summarize the types of functional differences that can occur within a species, and we describe the evolutionary and ecological processes that shape intra-species diversity, including within an individual host. Finally, we survey within-host intra-species diversity across different sites in the human body, and we ask how within-host intra-species diversity affects the functional role of the human microbiota and the outcome of infection.

## TERMINOLOGY OF INTRA-SPECIES DIVERSITY

To study intra-species diversity, it is first necessary to define a species. Bacteria can be compared using phenotypic or genomic differences. Phenotypically, species are often defined as exhibiting unique traits, for instance related to growth, metabolism, or morphology. Genotypically, small-scale differences across a set of genomes can be compared by assessing the number of point mutations; these are all termed single-nucleotide variants (SNVs) and can also be called single-nucleotide polymorphisms (SNPs) when present in more than 1% of the population (5). At a larger scale, genomic differences between bacteria can be defined using average nucleotide identity (ANI), which quantifies the sequence conservation across shared portions of the genome (6). Bacteria within the same species usually have at least 95% ANI (7, 8). In addition, researchers consider differences in gene content across bacteria (9), which often scale with ANI differences (10), but not always (11, 12). Alternatively, in amplicon-based sequencing analyses of the bacterial 16S rRNA gene, a species is usually defined as having 99% sequence similarity, but this can vary across different genera (13, 14).

Genomic diversity below the species level is referred to by many terms, including subspecies, microdiversity, population, lineage, and strain (Table 1) (5, 15, 16). Terminology is not used consistently, obscuring some of the insights within this field (15). Some of this inconsistency comes from different techniques and perspectives used to differentiate bacteria (e.g., phenotypic vs genomic approaches). For example, phenotype-based classifications include terms such as ecotypes (ecovars), pathotypes (pathovars), phagotypes (phagovars), and serotypes (serovars), which distinguish bacteria based on functional roles, pathogenesis, phage susceptibility, or surface antigens, respectively (15, 16). In contrast, genomovar is a genome-based definition that only considers ANI and gene content differences (10, 17, 18). Other terms vary across studies. For example, lineage can be used broadly to describe any bacterial taxon, but within the intra-species diversity field, it is used specifically to describe bacterial diversification within a single person, often resulting in less than 100 SNVs (5, 19, 20). Strain is especially variable in usage, where it sometimes is synonymous with isolate or clone, but other times used to describe bacteria with greater than 99.99% ANI based on sequencing analysis (10, 17, 21).

**TABLE 1:** Subset of terms used for intra-species variation, generally organized from least diverse to most diverse

Term	Definition(s)	Genetic diversity	Reference(s)
Isolate, individual, or clone	Genetically identical cells	100% ANI	
Strain	A single culture or subcultures of it, synonymous with isolate; often used with culture-based approaches	No, or minimal, differences	(21)
	A group of isolates with high genomic similarity; often used with sequencing approaches	>99.99% ANI, gene content >99%	(10, 17)
Lineage	A group of bacteria that has diversified within a single person	Usually less than 100 point mutations	(19, 20)
Genomovar	Genome-based intra-species units; likely includes multiple strains with different phenotypes	~99.5% ANI, gene content >90%	(10, 17, 18)
Population	Set of individuals of the same species with significant gene flow	>95% ANI	(22, 23)
Subspecies	Groups of strains with disrupted gene flow, potentially undergoing speciation	>95% ANI	(15)

Here, we use the terms intra-species diversity, microdiversity, and population diversity to broadly describe the idea of within-species differences. In addition, as we review the literature, we note the different approaches authors use to distinguish genotypes and the level of diversity observed.

## STUDYING WITHIN-HOST MICRODIVERSITY

### Approaches to measure within-host microdiversity

Our ability to characterize within-host microdiversity has been fueled by the expansion of sequencing technologies. Some studies use 16S rRNA gene sequencing to measure different sequence variants and assess population-level diversity (24, 25). Furthermore, full-length 16S rRNA gene sequencing or ribosomal 16S–23S intergenic spacer region sequencing can increase the resolution to resolve strains (14, 26). Currently, a common approach for assessing microdiversity is metagenomics, which often measures microdiversity by comparing sequencing reads to some sort of reference sequence. Sometimes, researchers use a marker gene database or an isolate genome as the reference sequence, but these references may lack representative sequences from novel taxa. Alternatively, reads can be aligned to a metagenome-assembled genome (MAG) as the reference sequence for novel taxa, but this approach may miss MAGs from rare strains and is more dependent on sequencing depth and MAG quality (27). While metagenomic sequencing is increasingly accessible, it can underestimate microdiversity at lower sequencing depths (28). Furthermore, variation in computational programs used to delineate strains using metagenomics results in inconsistent estimates of microdiversity across studies.

Beyond directly sequencing from samples, other studies will culture multiple isolates of the same species from a single sample and then measure microdiversity using phenotypic comparisons or by sequencing the genomes of the isolates, often using a high-throughput approach (29). Isolate genomes can be compared with techniques such as ANI, pangenomics, or phylogenomics. Isolation-based approaches are advantageous to be able to compare across the full genome of an organism, which can be challenging in metagenomics. Furthermore, an additional strength of isolation approaches is the ability to directly connect sequence diversity with functional (phenotypic) differences. However, isolation approaches are difficult to scale up, and cultivation challenges can limit the taxa that can be studied in this manner and introduce biases (17). To overcome cultivation challenges, there is also the potential to use single-cell genomics (30–34), but single-cell genomes are often lower quality.

For all these approaches, a primary limitation is resolving the functional implications of the sequence diversity, except where isolates can be characterized experimentally. Thus, it can be powerful to combine metagenomic and isolation approaches (11, 19, 35). Another challenge with existing approaches—both sequencing and culture-based—is the lack of quantification. While these methods can identify and assess strain-level diversity, they cannot provide robust quantitative measures of strain abundance. Metagenomic approaches often do not have enough coverage of strains within a sample to assess abundance (36), and culture-based approaches can be biased toward less dominant strains (17).

### Spatial and niche considerations

In quantifying within-host microdiversity, it is important to consider how different genotypes are arranged across space within a body site. In the gut, oral cavity, skin, and other microbiome sites, there are complex host structures that can separate bacteria, such as crypts, subgingival pockets, and pores, and areas where bacteria are thought to be more mixed, such as the gut lumen (37). A bacterial cell with a volume of 1 µm^3^ is infinitesimal in size compared to these structures (38). Furthermore, different environmental factors can permit or restrict the growth of a microbe. The theoretical multidimensional combination of environmental factors that permit growth of an organism defines its fundamental niche, and the environments, interactions, and conditions that permit growth of an organism *in situ* define its realized niche (39). Applying this concept to the human microbiome, we can think about the Baas Becking hypothesis that “everything is everywhere, but the environment selects”; a species’ fundamental niche may be everywhere across a body site (e.g., gut), but the differences in the physical, nutritional, and immunological environment within a site (e.g., lumen vs crypt) shape the realized niche and, thus, the genotype(s) and functions of the species present (40). For example, mucoid strains of *Pseudomonas aeruginosa* can be selected for in the cystic fibrosis lung as a mechanism to protect against the host immune response (41).

Given this complexity, it is important to consider the spatial scale captured by studies of within-host microdiversity. When multiple variants of a bacterial species are found within a human site or sample, it can be challenging to know if variants are spatially or functionally isolated from each other. Most microbial interactions occur on the scale of 10–100 µm (42, 43), but many of the current approaches may not adequately capture the scale at which these interactions occur or consider the functional or realized niches of genotypes. For example, spatial and niche specialization has been observed in *Cutibacterium acnes*, where horizontal gene transfer has driven specialization to different parts of the skin (forearm, face, or antecubital fossa) as well as different disease states (health, atopic dermatitis, or acne) (44). Additionally, at the micron scale, strain separation across skin pores can promote the coexistence of multiple lineages of *C. acnes* within a small patch of skin (45). In the oral microbiome, different *Haemophilus parainfluenzae* strains inhabit specific niches, such as the supragingival plaque, buccal mucosa, and tongue dorsum (46), and these different strains possess unique gene clusters (47). In contrast to the skin or oral microbiome, it is difficult to sample the gut, and fecal samples reduce the meters of niches within the gastrointestinal tract into a single composite sample. This challenge has been addressed with *in vivo* and *in vitro* model systems, which have identified different strains within the lumen and mucosal surfaces and spatial segregation between glands and crypts (48–50), but not along the length of the gut (51).

## FUNCTIONAL DIFFERENCES WITHIN SPECIES

Our understanding of within-host microdiversity is significantly shaped by the vast work to characterize the types of functions that can vary across a species, both defined through phenotypic analyses and inferred through comparative genomics. Here, we highlight some key differences that vary within species that impact human health (also reviewed elsewhere [52, 53]).

Within a species, there are not usually broad differences in central metabolism (e.g., autotrophs vs heterotrophs) or growth rate optima (52, 54). However, many microbes exhibit intra-species differences in nutrient assimilation and metabolic outputs. For example, *Bifidobacterium longum*, a gut mutualist, consists of multiple subspecies that are found at different stages of life and degrade different carbon sources (55). *B. longum infantis* is found in the infant gut and can use human milk oligosaccharides (HMOs) as a carbon source and urea, which is highly prevalent in human milk, as a nitrogen source (56–60). In contrast, *B. longum longum,* which lacks HMO- and urea-utilization genes, typically replaces *Bl infantis* in the first few years of life (57). Similarly, *Bacteroides fragilis* strains in the gut can vary in starch import, altering their role as a member of the gut microbiota (61, 62), and a number of *Bacteroides* and *Parabacteroides* species display population-level diversity in binding to dietary glycans (63). These differences in the uptake and utilization of nutrients across species can also have other impacts on host health and the microbiota. For instance, variation has been shown in the ability of *Eggerthella lenta*, a member of the human gut microbiota, to metabolize drugs, altering drug efficacy and toxicity (64).

Intra-species differences in virulence pathways can delineate a health-associated microbe from a pathogenic strain (65–68). One way that virulence can vary across strains is through the presence of pathogenicity islands, large horizontally transferred clusters of genes that encode virulence factors. These islands were first identified in *E. coli* in 1990 (69) but have since been described in a number of pathogens, including *Staphylococcus aureus* (70) and *Listeria monocytogenes* (71). For *E. coli*, these islands can confer pathogenicity and even determine which tissues the strain will infect, such as for uropathogenic *E. coli*, where pathogenicity islands allow for binding to sugar moieties on epithelial cells in the renal tract and kidneys (72, 73). Beyond a full pathogenicity island, differences in specific toxin genes can also influence disease outcomes. For *Clostridioides difficile*, a major cause of diarrheagenic illness, pathogenicity requires complete versions of at least one cytotoxin gene in the genome (74–76). In addition*,* other hypervirulent *C. difficile* strains produce a second binary ADP-ribosyltransferase toxin (77, 78).

Furthermore, within a species, many human-associated bacteria vary in their ability to engage with the immune system and elicit an immune response. This variation often arises from intra-species diversity in surface structures (e.g., capsule presence and composition, resulting in different serotypes), as well as in secreted toxins (79, 80). *Streptococcus pneumoniae* has over 100 serotypes with different polysaccharide capsules and extracellular proteins (81, 82). Diversity within the capsule is thought to benefit pneumococci by protecting strains from recognition by the adaptive immune response. Even members of the same capsular serotype can differ in proteins that inhibit the complement system (83). Similar diversity in serotypes can occur across many bacteria, such as in *B. fragilis* and *Bacteroides thetaiotaomicron,* which produce distinct polysaccharide capsules (84). In *Bacteroides* species, this capsule variation drives differences in immunomodulation and biofilm formation (85, 86). Beyond capsule differences, bacterial species can also vary in other ways that alter their immune interactions. For example, biofilm formation can vary within a species (87–89), with the extracellular polymeric substances that protect biofilms altering immune detection and killing (90).

Finally, antimicrobial resistance can vary substantially across strains, with serious implications for host health. Strain diversity in antimicrobial responses is often driven by geography and by the past history of antimicrobial exposure (91, 92). Most pathogens exhibit some level of heterogeneity in antimicrobial resistance, including *C. difficile*, *S. aureus*, *S. pneumoniae*, *E. coli*, and *Klebsiella pneumoniae* (93–97).

## PATTERNS AND PROCESSES OF WITHIN-HOST INTRA-SPECIES DIVERSITY

### Generation, maintenance, and depletion of microdiversity

**Fig 1:**
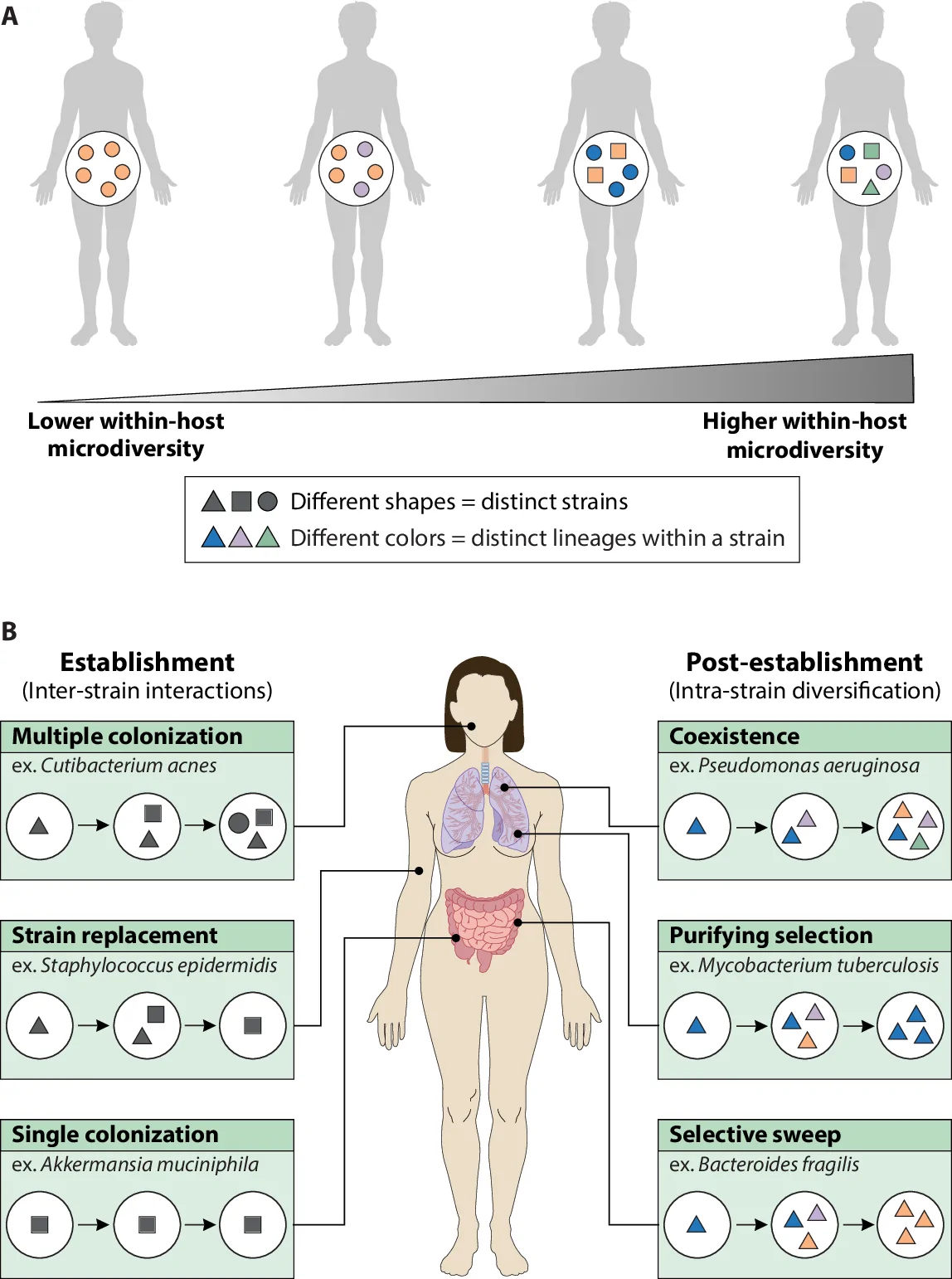
(**A**) Within-host microdiversity. Individuals can harbor a single strain or multiple strains of a species, represented by the different shapes. Furthermore, each strain can vary from having a small number of SNPs (represented by shapes with the same color) to having high genotypic diversity (represented by different colors). (**B**) Processes and patterns of within-host microdiversity across the human body. Microdiversity occurs across the body in both bacteria associated with health, such as *Staphylococcus epidermidis* on the skin (19), and bacteria associated with disease, such as *Pseudomonas aeruginosa* in the lung (106, 107). Microdiversity is shaped by the number of independent strains that establish within a body site (different shapes) and through the diversification of these strains (different colors). Species examples for each process represent common trends, but other patterns are also observed. For example, selective sweeps have also been observed for *P. aeruginosa* in the lung (108). Furthermore, the spatial scale can alter the interpretation of microdiversity, such as for *Cutibacterium acnes* where the establishment of single strains within pores allows for the establishment of multiple strains across pores (45). Citations for all examples can be found in the main text and in Data set S1.

In the human body, the level of intra-species diversity for any single site for a particular species can vary (Fig. 1A). However, most studies that have looked for within-host microdiversity, both for members of the microbiota and for pathogens, have found some level of genomic heterogeneity, often with one to five lineages or strains per site (25, 98, 99). To understand the underlying mechanisms that alter this microdiversity, populations have been tracked in humans and mouse models over time (35, 100, 101). Each species within each person has a unique ecological and evolutionary history (102), shaped by mutation, horizontal gene transfer, drift, and migration. Thus, broadly, the level of microdiversity within a body site will be defined by the number of strains that establish and persist at any one time and the level of diversification that occurs after establishment (Fig. 1B and Data set S1, reviewed in detail elsewhere [103–105], and summarized here).

New strains are introduced into the microbiota via migration (109). Often, migration is observed within families and household contacts. For example, strains can be shared between parents and children, both for pathogens and for members of the healthy microbiota (19, 110–114). Generally, the highest level of intra-species diversity occurs when distinct strains are established via multiple colonization events and then coexist (Fig. 1B). For example, this has been observed for *C. acnes* (19). Alternatively, one strain may dominate in a site and prevent other strains from colonizing, leading to low microdiversity, as is the case for *Akkermansia muciniphila* in the gut (115, 116). More commonly, a single strain will dominate at a time, but turnover between strains will occur as new strains establish and replace the original strain. This pattern has been described in *Staphylococcus epidermidis* on the skin, *B. longum* in the infant gut, and *S. pneumoniae* in the nasopharynx (19, 60, 117).

**Fig 2:**
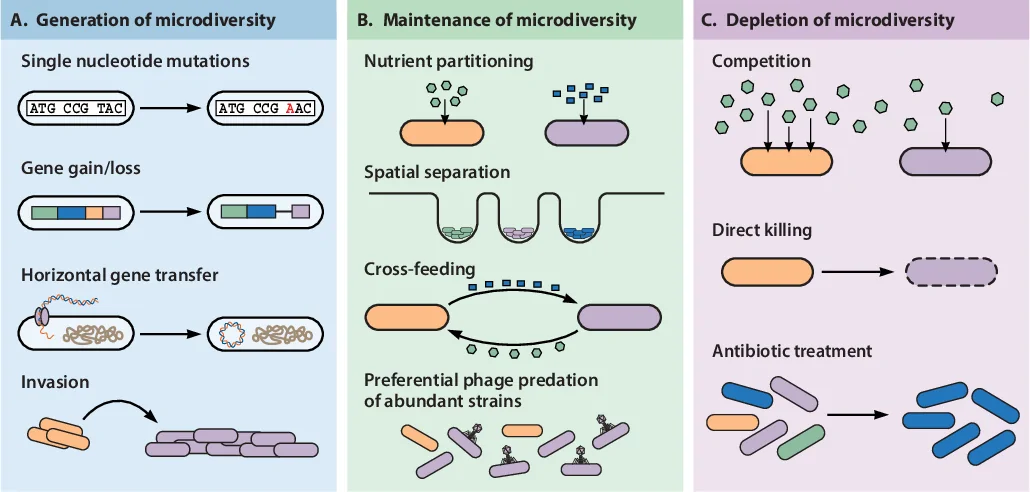
The generation (**A**), maintenance (**B**), and depletion (**C**) of microdiversity are altered by evolutionary and ecological processes.

After establishment, a strain may diversify or maintain overall low sequence variation (Fig. 1B and Fig. 2A). As bacteria experience 10^−5^ to 10^−10^ mutations per generation (118, 119), genomic diversity is created when bacteria replicate their DNA and divide. Single mutations can have a substantial impact on microbial fitness, and the population-level abundance of a mutation may be driven by selection. For example, in *Streptococcus suis*, a single amino acid substitution in Penicillin Binding Protein 1a is able to confer resistance to penicillin and amoxicillin in otherwise identical strains (120). Alternatively, changes in mutation abundance may be driven by neutral processes, such as drift, especially for taxa with low population sizes (105, 121). In chronic infections, bacteria can evolve to inactivate DNA repair mechanisms, becoming hypermutators and allowing them to quickly accumulate both adaptive and non-adaptive mutations (122–125). Gene duplication and deletion can also lead to differences in gene copy number between strains, eventually resulting in new gene variants as duplicated genes diverge over evolutionary timescales (126, 127). Additionally, over evolutionary time, lateral gene transfer is often even more common than mutations (128, 129). For example, antimicrobial resistance can be transferred through acquiring plasmids or prophage (130, 131), and lateral gene transfer is a major driver of *P. aeruginosa* diversification in people with cystic fibrosis (pwCF) (132). In contrast, recombination from lateral gene transfer can also promote genetic similarity within a population by removing novel variants (133).

Together, post-establishment diversification processes can manifest into different patterns of microdiversity (Fig. 1B). For instance, microdiversity is generated for *P. aeruginosa* in the lungs (91, 134), potentially driven by selective or neutral processes (105, 108). In contrast, purifying selection removes deleterious mutations, maintaining a low microdiversity, for example, in chronic *Mycobacterium tuberculosis* infections (91, 134). Alternatively, selective sweeps have been observed for many species in the gut, including *B. fragilis*, in which a newly evolved allele becomes dominant over time, also maintaining low intra-species diversity (35, 135–137).

Establishment and post-establishment dynamics work in tandem. For example, *S. pneumoniae* in the nares not only exhibits strain replacement but also experiences purifying selection once established (117). Multiple unrelated *C. acnes* strains can establish and coexist on the skin, and then each strain goes on to further diversify into its own lineage (19). In the human gut, *de novo* mutation and recombination events are thought to shape diversity over shorter time periods (i.e., months), while invasion of new, distinct strains shapes microdiversity in the long term (i.e., decades) (135). Additionally, the unique biological context of each host and each bacterial species can result in different patterns and processes across people. For example, while *B. fragilis* almost always maintains a low microdiversity via selective sweeps in the gut, one study observed stable coexistence of two sublineages in one person (35). Here, a sublineage with an advantageous glycosyltransferase mutation coexisted with a second sublineage, driven by prophage-mediated killing of the sublineage with the glycosyltransferase mutation.

Furthermore, although human microbiome sites and infections are constantly exposed to novel genotypes of bacteria, through either mutation or migration, not all novel genotypes are able to persist in the microbiome over time. Experimental evolution studies *in vitro* have shaped our understanding of how new mutations can establish within a population. For example, in the long-term evolution experiment with *E. coli*, a subpopulation capable of using citrate as a novel carbon source evolved and persisted with the ancestral cells that could not use citrate (138). However, this innovation only evolved after over 30,000 generations and required specific potentiating mutations. Although these types of *in vitro* experiments do not capture the complexity of the host environment, they demonstrate many of the processes that influence strain dynamics, including historical contingencies, frequency-dependent selection, and predictability in evolution (139, 140).

### Role of interactions in the maintenance or depletion of microdiversity

Beyond observing the generation of diversity, it is important to understand the ecological and evolutionary processes that shape how much microdiversity is maintained in a population within an individual host (Fig. 2B and C) (102). In particular, microbial interactions are a key force in modulating microdiversity through altering the realized niche of different variants.

One type of interaction that can influence microdiversity is interactions among genotypes within a species (intra-species interactions). Based on the competitive exclusion theory in ecology, if two strains occupy a similar niche, both will not persist long term (141). Yet, multiple strains of the same species have been observed to coexist (15, 46, 142). Although this coexistence may be an artifact of sampling limitations (see “Spatial and niche considerations,” above), it may also be shaped by differences in the realized niche or by direct interactions. For example, nutrient use or availability often governs the maintenance of microdiversity within host microbiome sites. Generalist organisms with more flexible or expansive metabolisms, such as *E. coli* or *B. longum*, are more likely to have multiple strains within a microbiome than specialist organisms that consume a particular nutrient, such as *A. muciniphila* (115). For organisms with variable metabolism, cooperative interactions, such as cross-feeding, can promote microdiversity. Persistence of multiple strains of *E. coli* and lactic acid bacteria has been observed in simulations and in *in vitro* synthetic community experiments when cross-feeding occurs (143, 144). In contrast, for organisms with narrow metabolic inputs, such as *A. muciniphila*, successful establishment is often stochastically determined by which strain colonizes first (116). Here, using a mouse model, the authors showed that a novel *A. muciniphila* strain could not establish in the gut microbiome if another *A. muciniphila* strain was already present (116). In addition, the chance of strain displacement is increased when the invading strain has unique nutritional needs compared to the resident strain (145). Beyond metabolism-driven interactions, antagonistic interactions within species, such as through the production of antimicrobial peptides (bacteriocins), are correlated with higher microdiversity of mutualist bacteria (146, 147). In contrast, for *S. epidermidis* and *B. fragilis*, intra-species warfare may prevent coexistence of certain strains (148, 149).

Inter-species interactions with other bacteria or viruses can also alter microdiversity within a host. In the human gut, bacterial species initially accumulate population-level diversity when communities are diverse (species-rich), but in the long term, lower intra-species diversity is observed in more species-rich communities, indicating that community diversity interplays with microdiversity in complex ways (150). Community diversity may decrease the number of niches available, blocking new strains from establishing within a population (145, 151). Also, genome-barcoded lineages of *B. thetaiotaomicron* were used to demonstrate that migration of genotypes was slowed when mice had a diverse gut microbial community (152). In the cystic fibrosis lung, higher within-lung *S. aureus* phenotypic diversity was associated with increased prevalence of *P. aeruginosa* (153). This finding potentially indicates that *S. aureus* phenotypic diversity promotes the coexistence of these two antagonistic bacteria, or it is possible that staphylococcal diversity and *P. aeruginosa* infection are both correlative signatures of a more progressed lung infection. Intra-species dynamics can also be driven by predatory interactions between bacteriophages and their bacterial hosts, which not only promote novel strains via mutation but also ensure the persistence of low abundance variants (35). The evolution of resistance mechanisms to phage can be a significant source of microdiversity (154). Additionally, since more abundant bacteria are more likely to become infected with a phage, negative frequency-dependent selection can occur, where the most abundant strain is selected against (155).

Finally, host-microbe interactions can alter intra-species diversity. For example, in pwCF, the initial colonizing population of *P. aeruginosa* can evolve over years into distinct sublineages with unique functional capabilities, such as antibiotic resistance or hypermutator phenotypes, spurred by environmental conditions specific to the cystic fibrosis lung, like oxidative stress and osmotic stress (41, 106, 156). The physical space provided by the host can also drive strain partitioning (see “Spatial and niche considerations,” above). For instance, a person’s sebaceous skin often is colonized with multiple *C. acnes* strains. The maintenance of *C. acnes* microdiversity is a result of spatial segregation, where each individual pore is colonized by a unique *C. acnes* strain (45). Spatial heterogeneity and isolation can also support multiple distinct lineages of *M. tuberculosis* or *P. aeruginosa* in the lung (106, 134). Although strains are universally distributed across the mouse gut, it is still possible that for species with within-host microdiversity, strains spatially segregate at a narrower spatial level, for instance across different crypts or between the lumen and mucosa (49, 157).

It is also hypothesized that the host may play a direct role in controlling their intra-species diversity (158). The host controls bacterial colonization through interactions with the immune system and by providing nutrients (e.g., mucins) (57, 83, 159), and intra-species diversity can occur both in a strain’s inflammatory properties and metabolic capacity. Although we understand little about how the host has evolved to promote high or low intra-species diversity within a system, one example of host influence on intra-species diversity is *B. fragilis* in the gut. *B. fragilis* expresses a specific capsule that can bind to immunoglobulin A, but only in the presence of host-provided mucin (160). This interaction allows only a single lineage of *B. fragilis* to penetrate the mucus and colonize the crypts (49). In addition, host behavior can shape microdiversity. Antibiotic use can remove diversity by selecting for specific variants, at least temporarily (161). Furthermore, strain diversity was higher over time in mice fed a diet with microbiota-accessible carbohydrates (152), showing the impact of diet on gut microdiversity.

## SURVEY OF WITHIN-HOST INTRA-SPECIES DIVERSITY

Given the large number of species found on the human body, across a number of different sites, intra-species diversity has only been defined in select cases. Furthermore, comparing intra-species diversity across sites and across bacterial species is challenging given that studies use different methods and different definitions to measure diversity. For instance, more variants are likely to be detected with more sequencing depth or isolation effort (17, 28). Counting the number of variants detected also does not provide insight into whether the variants are distributed equally in abundance (evenness) or if a specific variant is dominant although these factors are important for the biology of the population. Below, we survey within-host intra-species diversity across different body sites. As possible, we describe the methods used to delineate genotypes, and additional information can be found in Data set S1.

### Gut microbiome

Within-host microdiversity is most studied in the gut. However, much of what we understand about gut microdiversity is across the full intestinal tract and does not account for niche or spatial segregation of genotypes. A person’s gut microbiome is typically co-colonized by between one and four strains of many abundant species (11, 98, 135, 162) though different individuals vary in their level of microdiversity (20, 163). Often, microdiversity is stable over time, but there are also instances where researchers have observed fluctuations (11, 35, 135, 164, 165).

Using a <99.95% ANI cutoff to distinguish strains, Chen-Liaw et al. described within-host intra-species diversity for 92 common gut microbes by isolating and sequencing bacteria from stool samples of 100 people (54 healthy and 46 with an intestinal disease) (98). Species with the most strains per person (mean) included *Turicibacter sanguinis* (2.57)*, K. pneumoniae* (2.33), *Streptococcus parasanguinis* (2.13), and *Blautia wexlerae* (2.06). Several commonly studied species of interest had an intermediate number of strains per person, including *Clostridium perfringens* (1.82), *B. longum* (1.75), *Enterococcus faecium* (1.63), *Eggerthella lenta* (1.60), *E. coli* (1.57), *Enterococcus faecalis* (1.55), and *Bifidobacterium adolescentis* (1.53). Other well-known species were present at an average of just one strain per person, including *Alistipes finegoldii*, *B. fragilis*, *Bacteroides intestinalis*, *Bacteroides massiliensis*, *Bifidobacterium breve*, and *Lactobacillus plantarum*. In particular, the low within-host microdiversity of *B. fragilis,* a long-term gut resident, has been further investigated (<100 SNPs per person, according to reference 35). For *B. fragilis*, low intraspecies diversity is maintained by intra-species warfare, host-microbe interactions, and occasional selective sweeps (Fig. 1B) (35, 149). Similarly, as discussed above, low intraspecies diversity of *A. muciniphila* is maintained by competitive exclusion of novel strains (Fig. 1B) (116).

These findings highlight that intra-species diversity and dynamics in the gut are not equal across species and can even vary within members of the same genus (98, 162, 163). This variation was further shown by Poyet et al., who used SNP distances to define strains for *Bacteroides vulgatus* and *Bacteroides ovatus* (11). Distantly related *B. vulgatus* strains with large SNP differences co-colonized individuals, and in one person, these strains coexisted at stable relative abundances for over 1 year. In contrast, three closely related *B. ovatus* genotypes coexisted in a different host, but the small SNP distances between isolates suggested a single colonization event followed by within-host diversification (11).

### Vaginal microbiome

An optimal vaginal microbiome typically has low species-level diversity and is dominated by a single species of *Lactobacillus* (166), providing a unique system to study intra-species diversity. Investigations of vaginal within-host microdiversity have primarily used metagenomic methods. Although different authors define “strain” differently, the literature suggests that common vaginal species exhibit within-host microdiversity. For instance, an analysis of *Lactobacillus crispatus*-dominated communities suggested each contained 1–20 *L. crispatus* genotypes at one time (167).

As in the gut microbiome, different species of vaginal bacteria exhibit different levels of within-host microdiversity (12, 168, 169). One study compared the number of nonredundant genes belonging to a particular species in a host’s metagenome to the number of nonredundant genes in the genome of a single isolate (168). Across hosts, the number of nonredundant genes belonging to a particular species in a person’s metagenome was almost always significantly higher than the number of genes in a single bacterium’s genome, indicating that distinct members of the species with different accessory genomes co-colonized at a given time. This pattern was found for *L. crispatus*, *Lactobacillus iners*, *Lactobacillus jensenii*, *Lactobacillus gasseri*, *Gardnerella vaginalis*, and *Fannyhessea vaginae*, but not for *Prevotella timonensis*, indicating low within-host diversity for this species. The ratio of metagenome to genome content was particularly high for *G. vaginalis* and *F. vaginae,* suggesting that these two species might have higher within-host diversity than the lactobacilli (168). Indeed, another paper that classified “strains” by co-occurring SNVs in the metagenome reported that vaginal samples containing *G. vaginalis* or *F. vaginae* were colonized by multiple strains 63% and 77% of the time, respectively (169). This finding was in contrast to the vaginal lactobacilli, which, when present, had multiple strains in 21% (*L. crispatus*), 19% (*L. iners*), 3% (*L. gasseri*)*,* or 2% (*L. jensenii*) of samples.

Preliminary evidence suggests the relative abundance of vaginal strains may shift over time. A longitudinal study of 10 women over 1–2 years detected persistent strain colonization in 6 women and strain turnover in 4 women, as defined as <99.999% ANI using metagenomics (170). In addition, in a cohort of 48 pregnant women, 27% of women did not have a change in the dominant vaginal microbiome species but did have a change in the dominant subspecies between trimesters 2 and 3, based on accessory gene content from metagenomes, suggesting either multiple colonization events over the course of the study or that baseline intra-species diversity existed below a detectable threshold at the initial sampling (12).

### Nasopharynx microbiome

Patterns of multiple genotypes are also present in the nasopharynx, which has been primarily studied for *S. pneumoniae* and *S. aureus*. In the nares, a person may be colonized by two or more *S. pneumoniae* serotypes at any given time (117, 171). However, long-term co-colonization with the same two serotypes is rare, indicating that strain turnover occurs (117). In the absence of antibiotic treatment, susceptible strains typically outcompete resistant strains (117, 172). There is also evidence that purifying selection acts on *S. pneumoniae*, especially on certain stress-response genes, making the population more homogeneous over time (117). For *S. aureus*, while not all people are colonized in the nares, some people may harbor more than one genotype simultaneously (173, 174). In a cohort of children with community-acquired MRSA infections, 67% were coinfected with more than one genotype in the nares, determined by repetitive PCR typing (174). Furthermore, a study of 360 asymptomatic adults carrying *S. aureus* in the nares demonstrated that even within a multi-locus sequence type group, *S. aureus* isolates from a single host contain unique SNVs and indels (175). *S. aureus* in the nasopharynx increases its microdiversity via introduction of diverse strains (176) but also evolves in the host via purifying selection on short timescales (175).

### Skin microbiome

On the skin, within-host intra-species diversity has been well-described for two dominant and abundant taxa: *C. acnes* and *S. epidermidis*. While these two taxa can both be strain diverse, the mechanisms governing the strain dynamics can vary (Fig. 1B). *C. acnes* strain colonization follows the priority effect, where the initial colonizer dominates a single pore, but different strains can colonize different pores (19, 45, 177). Thus, over time, the *C. acnes* strains within a pore and across pores can be remarkably stable (19, 45, 177). This within-a-pore isolation may also contribute to the evolution and specialization of different *C. acnes* strains to different sites on the body, such as the face or forearm (44). *S. epidermidis* strain populations can undergo frequent turnover (19), and strains are often transmitted between different parts of the body (178). Other work has also observed strain sharing and replacement dynamics for ESKAPE (*Enterococcus faecium*, *S. aureus*, *Klebsiella pneumoniae*, *Acinetobacter baumannii*, *P. aeruginosa*, and *Enterobacter* species) pathogens on the skin (179).

### Oral microbiome

In the oral microbiome, within-host strain diversity has been characterized, although not as in-depth as other microbiome sites. Within-host strain diversity has been detected in saliva for *Streptococcus sanguinis*, *Streptococcus parasanguinis*, and *Rothia mucilaginosa* (180). However, saliva does not resolve if these different strains partition to different niches within the mouth, such as tongue vs supragingival plaque (181). When researchers focus on individual sites within the oral cavity, they have identified high levels of microdiversity for some taxa but not others, with considerable variation across individuals (25, 26, 99, 112, 182, 183). Despite these studies highlighting within-host microdiversity for several oral bacteria, further work is needed to investigate the mechanisms underlying the strain-level dynamics.

### Acute and chronic infections

Polyclonal infections are estimated to make up 11% (median) of all infections, with at least 21 bacterial pathogens reported to exhibit high microdiversity within infections (184). Strain diversity (as determined by multi-locus sequence typing) has been observed for bacteria in acute infections in rare cases, such as diarrheagenic *E. coli*, likely driven by introduction of multiple genotypes (185). However, pathogens initiating most acute infections are reported to have low within-host diversity, such as for *Bordetella pertussis* in patients with whooping cough, where the majority of isolates from a single individual had no SNPs (186, 187).

In contrast, in chronic infections, such as chronic lung infections, chronic wounds, and chronic urinary tract infections (UTIs), pathogens are present for longer times, increasing the opportunity for diversification, as well as leading to significant morbidity and mortality (188). Diversity of bacteria within the lungs of pwCF is highly researched, where bacteria can persist over decades and contribute to a decline in lung function (189). Longitudinal studies of the lungs of pwCF reveal that *P. aeruginosa* may colonize multiple times, and strains can coexist for decades after chronic infection has been established (107, 190, 191). Post-establishment, these lineages will continue to diversify (Fig. 1B) (108, 190). However, another study observed rapid *P. aeruginosa* strain turnover rather than stable co-colonization in pwCF despite high *P. aeruginosa* microdiversity as determined by differences in phenotypic and genotypic traits (192). Interestingly, clonal *P. aeruginosa* infections may establish in different regions of the lung, experience different selective pressures in those regions, and eventually diversify into sublineages with different morphologies (106). Other bacteria found in the lungs of pwCF exhibit little diversification. In one study, *Burkholderia dolosa* isolates varied by no more than 20 SNVs across the whole genome although certain mutations enhanced colonization in long-term infections (193). Diversification has also been studied for other species, such as *S. aureus*, *Burkholderia cenocepacia*, and *Mycobacterium abscessus*, in the lungs of pwCF, highlighting overall low microdiversity but also multiple co-existing lineages of *S. aureus* in some pwCF (194–197).

Within-host microdiversity has been described by a handful of studies for other chronic infections but remains understudied for chronic wounds, chronic UTIs, and many other infections such as recurrent otitis media. For chronic tuberculosis, *M. tuberculosis* can persist over years in the lungs. A single colonization event is typical though a minority of people are colonized multiple times facilitated potentially by spatial separation across the lung (134, 198). Lineages may then diversify, and variants can coexist stably (134, 198). This diversification is usually minimal, and over time purifying selection can remove novel variants from the population (91, 134); one study recorded a maximum of just 59 SNVs across within-host lineages (134). On the skin of people with atopic dermatitis, *S. aureus* colonization is usually driven by a single genotype that diversifies over time (typically by <40 SNVs), and atopic dermatitis flares can be caused by blooms of individual strains (199, 200). In the stomach, *Helicobacter pylori* can persist for years, but the level of and origins of its microdiversity are disputed. Some amplicon typing studies have found up to six genotypes of *H. pylori* that coexist in the stomach of infected individuals, with higher diversity associated with more severe disease outcomes (201, 202). Due to the high conservation among key housekeeping genes in these strains, authors suggested that these patients were likely initially infected by a single genotype of *H. pylori,* which diversified over the course of the infection (201). In contrast, another study reported all participants harboring just one clonal type (as defined by amplicon typing) of *H. pylori* (111), and it has also been shown in mice that *H. pylori* segregates single genotypes into individual glands in the stomach (48).

## IMPLICATIONS OF WITHIN-HOST MICRODIVERSITY FOR HUMAN HEALTH

**Fig 3:**
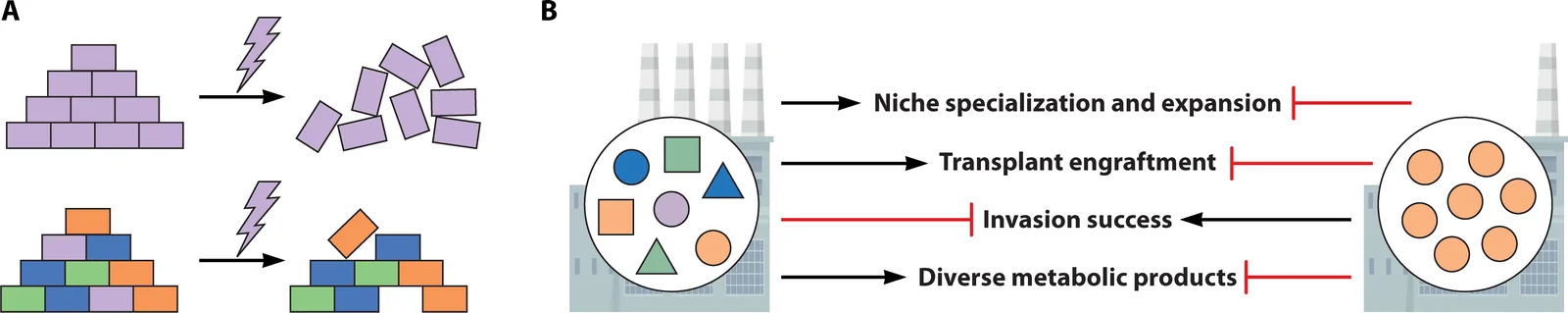
(**A**) Within-host population diversity can promote stability after disturbance**.** Each different colored block represents a different strain of a single species within a microbial community, where the top community is dominated by one strain and the bottom community is comprised of multiple strains. A disturbance, such as exposure to antibiotics, represented with the purple lightning bolt, can result in decreased abundance or death of certain cells (here, the purple strain). The loss of these cells results in a collapse of the less diverse community, whereas the more diverse community is disturbed, yet remains more intact. (**B**) Microbiomes, represented as factories, provide valuable functions to the host. Here, the microbiome factory on the left contains higher intra-species diversity compared to the microbiome factory on the right. The more diverse population can drive niche specialization, niche expansion, and the production of diverse metabolic products. Also, probiotics or microbiome transplants with higher microdiversity have increased success of engraftment. Conversely, higher microdiversity within a host can inhibit the invasion and establishment of novel strains, which can be beneficial in terms of inhibiting pathogens or harmful in terms of reducing microbiome transplant success.

The study of population diversity in bacteria, including within hosts, has primarily been examined based on the processes that result in microdiversity, as described above. However, we know considerably less about why within-host population diversity matters, including for host health. In macroecology, the impact of intra-species diversity within an ecosystem is also underexplored, but studies emphasize that population genetic diversity can increase productivity, stabilize population size, contribute to species coexistence, and alter predator-prey dynamics (203, 204). Additionally, ecological processes can be strongly impacted by variation in the level of intra-species diversity, often at a similar level as if the focal species is entirely removed from the ecosystem (205). In microbiology, it is reasonable to hypothesize that within-host microdiversity can have a similar impact on bacterial species and communities, and ultimately on their hosts, and here, we review examples of these relationships within the human microbiota and chronic infections (Fig. 3).

### Community stability

Within-host bacterial microdiversity can stabilize the microbiome and its functional capacity over time (Fig. 3A). The ecological theory underlying this relationship is the insurance hypothesis, whereby a more diverse community is more likely to have taxa or strains with a variety of traits (206). These traits enable resistance and resilience to disturbances, such as antibiotic treatment or pathogen invasion, preserving the function of the overall community (207, 208). Microdiversity within a bacterial population almost always will introduce additional functional capability and expand the niche space of a species, allowing it to engage with other microbes and the host in different ways (52). Based on clustering of amplicon sequencing, intra-species diversity has been observed to contribute to stability of taxa in freshwater lakes (24) and provide stability against viral infections in marine environments (209). Also, in *in vitro* experiments, biofilms containing more than two phenotypically distinct strains had increased tolerance to anionic stress (151).

### Antimicrobial responses

A common disturbance for microbiomes and chronic infections is antimicrobial exposure, and some studies have considered how microdiversity can alter responses to antimicrobials. For *P. aeruginosa* isolates sampled before and after antibiotic treatment from the lungs of pwCF, the presence of multiple strains, based on multilocus sequence typing, led to larger increases in antimicrobial resistance compared to low-diversity populations (210). The authors show that this increased resistance was driven by the selection of pre-existing resistant strains. However, the relationship between diversity and antimicrobial resistance can vary. In a separate study of *P. aeruginosa* isolates from the lungs of pwCF, within-host microdiversity, as determined by the number of SNPs, was correlated with diversity in antimicrobial resistance for some drugs, but not others (211). Also, in *in vitro* experiments with two-strain populations of a methicillin-susceptible and a methicillin-resistant *S. aureus* isolated from the lungs of pwCF, coculture did not impact the acquisition of resistance to oxacillin or vancomycin relative to monoculture (195). In addition to classical antibiotics, phenotypic diversity in *P. aeruginosa* has been shown to provide resistance to hydrogen peroxide (212).

### Probiotics and microbiome transplants

The insurance hypothesis also predicts that microdiversity will influence the establishment and persistence of new organisms within the microbiome. This relationship is of particular interest for probiotic formulation and microbiome transplantation, which have demonstrated encouraging results in select cases, particularly for persistent chronic infections or dysbioses, like bacterial vaginosis (213, 214) or *C. difficile* infections (215, 216). However, increased applicability of microbiome transplants or probiotics requires a better understanding of the factors that lead to the establishment and persistence of probiotic strains or the transplanted consortia. Some research has focused on the microdiversity of the introduced bacteria. For example, in a rat model, probiotics with multiple strains established more effectively than probiotics with single strains (217) although the use of fecal samples does not allow for investigation of the interactions that may govern strain displacement and establishment. Additionally, in a germ-free mouse model, strain mixtures of *B. ovatus* were more successful than individual strains in displacing resident *B. ovatus* strains (218). Also, a vaginal probiotic containing three strains of *L. crispatus* with diverse accessory genomes was effective in a human clinical trial (219). Similarly, fecal microbiome transplants with more strains (defined at >99.5% ANI) were more likely to colonize compared to transplanted communities with fewer strains due to differences in engraftment efficiency across strains (98). Fewer studies have considered how the population diversity of the native, resident microbiota impacts the success of microbiome transplant or probiotic outcomes, an idea often referred to as priority effects (220). In a model *in vitro* community, success of *Serratia* invasion decreased as the microdiversity of the resident *Pseudomonas fluorescens* increased (221). In mice, a resident microbiome with diverse strains competitively excluded transplanted strains from the same species (116). This antagonism could be mediated through multiple mechanisms, including directly via intra-species warfare and indirectly through phage inhibition, as more strain-diverse microbiomes can support more diverse phage communities (148, 149, 222). Together, these findings indicate that it may be important to consider the microdiversity of the native microbiota in ensuring the success of microbiome transplants or probiotics.

### Disease progression

Within-host microdiversity can directly impact host health, but conflicting theories have been proposed for the relationship between microdiversity and health outcomes. Increased strain diversity, which can produce additional antigenic epitopes, may lead to worse health outcomes by presenting a higher burden to the immune system. Alternatively, intra-species competition between pathogenic strains may reduce their burden and benefit the host (184). However, neither of these ideas have been thoroughly tested in bacteria. Several observational studies have detected a correlation between intra-species diversity and disease progression. For instance, increased nucleotide diversity of *Gardnerella* in the vaginal microbiome was associated with preterm birth, a major cause of maternal and neonatal morbidity (223). In pwCF, higher within-host *S. aureus* diversity in the lung, based on variation in the short sequence repeat region of the *spa* gene, was associated with increased inflammatory markers, including IL-6 (153), and higher *P. aeruginosa* diversity was associated with current lung disease severity and future lung function decline (224). While these studies are correlative, they indicate some relationship between within-host microdiversity and health. Controlled studies have the potential to complement these analyses. For instance, researchers observed increased pathogenicity in a two-strain population of *S. aureus* relative to monoinfection, likely due to intra-species interactions (225). Finally, most of this research focuses on microdiversity in pathogens, but within-host microdiversity in mutualistic members of the microbiota also likely impacts host health, potentially through promoting stability, as discussed above.

## CONCLUSIONS AND FUTURE DIRECTIONS

There is clear evidence that microbial species are diverse, often within a single person. Microdiversity exists for many taxa within the microbiome, where often people are colonized by a small number of strains. In addition, intra-species diversity can be measured in chronic infections. Further efforts to systematically quantify the diversity of bacteria across sites will aid in our understanding of bacterial dynamics in the host environment. In particular, we have a poor understanding of how the within-host intra-species diversity of the microbiota changes between health and disease states, for instance, in inflammatory bowel disease in the gut or periodontitis in the oral cavity. Furthermore, continued methodological advances can aid in more consistent approaches for classifying levels of microdiversity and the processes that shape the diversity of different taxa. It is also important for researchers to continue to assess the micron-scale spatial distributions and niche differences of genotypes in their consideration of within-host microdiversity.

We are just beginning to understand how this within-host microdiversity contributes to the ecology and evolution of the human microbiota and the development of disease. Select studies show that within-host microdiversity is an important factor in community stability, antimicrobial resistance, engraftment success of probiotics and microbiome transplants, and overall host health. Moving forward, there are vast opportunities to study the impact of within-host microdiversity both using defined consortia in experimental model systems and using thoughtfully designed clinical studies. A continued investment in studying the implications of harboring low or high intra-species diversity will provide insights into variation in disease outcomes across individuals, such as in chronic infections. These studies are also important for designing manipulations of the microbiota, including probiotics and microbiome transplant approaches. In addition, there is a potential to use vaccination approaches to alter strain diversity in the microbiota (226).

It will be important to consider that functional diversity exists beyond bacterial genomic microdiversity and beyond the human host. Similar levels of microdiversity exist for other host-associated organisms, including fungi, bacteriophage, and other viruses (227, 228). Intra-species microdiversity affects inter-kingdom interactions (229), but little is known about the implications of microdiversity in these taxa. In addition, beyond genomic diversity, organisms can also vary in their transcriptional activity and protein profiles, both within and between environments, adding further functional complexity to bacterial populations and communities. Finally, beyond human health, intra-species diversity also likely leads to similar dynamics in non-human hosts, such as other animals and plants, as well as within geographically restricted populations in the environment (17, 24), or even in wide-spread bacteria (33) or in the built environment (230). Thus, research in this area will broadly impact our understanding of bacterial dynamics across ecosystems.

## SUPPLEMENTAL MATERIAL

Data set S1 iai.00062-26-s0001.xlsx Studies defining microdiversity in the human body.

ASM does not own the copyrights to Supplemental Material that may be linked to, or accessed through, an article. The authors have granted ASM a non-exclusive, world-wide license to publish the Supplemental Material files. Please contact the corresponding author directly for reuse.
